# 免疫检查点TIGIT在肺癌免疫治疗中的研究进展

**DOI:** 10.3779/j.issn.1009-3419.2022.102.45

**Published:** 2022-11-20

**Authors:** 洁琼 吴, 敦强 任, 焕焕 毕, 冰倩 易, 红梅 王

**Affiliations:** 1 266000 青岛，青岛大学附属医院呼吸与危重症医学科 Department of Respiratory and Critical Care Medcine, The Affiliated Hospital of Qingdao University; 2 266000 青岛，青岛大学医学部 School of Medicine Qingdao University, Qingdao 266000, China

**Keywords:** TIGIT, PD-L1, 免疫治疗, 肺肿瘤, TIGIT, PD-L1, Immunotherapy, Lung neoplasms

## Abstract

T细胞免疫球蛋白和免疫受体酪氨酸抑制基序结构域（T cell immunoreceptor with immunoglobulin and immunoreceptor tyrosine-based inhibition motif domain, TIGIT）是近年新发现的免疫检查点分子，主要表达于T细胞及自然杀伤（natural killer, NK）细胞表面。通过与白细胞分化抗原155（cluster of differentiation 155, CD155）等配体结合，抑制T细胞及NK细胞介导的免疫反应影响肿瘤微环境。多项临床前研究已经证实，TIGIT/CD155通路可在多种实体肿瘤和血液系统肿瘤中发挥作用。目前研究TIGIT抑制剂单药或联合程序性死亡受体1（programmed cell death 1, PD-1）/程序性死亡受体-配体1（programmed cell death ligand 1, PD-L1）抑制剂治疗肺癌的临床试验正在进行中。

## Abstract (en)

T cell immunoreceptor with immunoglobulin and immunoreceptor tyrosine-based inhibition motif domain (TIGIT) is a newly discovered immune checkpoint molecule, mainly expressed on the surface of T cells and natural killer (NK) cells. By binding to cluster of differentiation 155 (CD155) and other ligands, it inhibits T cell and NK cell-mediated immune responses and affects the tumor microenvironment. Multiple preclinical studies have demonstrated that the TIGIT/CD155 pathway plays a role in a variety of solid and hematological tumors. Clinical trials investigating TIGIT inhibitors alone or in combination with programmed cell death 1 (PD-1)/programmed cell death ligand 1 (PD-L1) inhibitors for lung cancer are currently underway.

国际癌症机构发布的数据^[1]^显示，由于烟草致癌物暴露及空气污染加剧，肺癌已成为2020年癌症死亡的主要原因，约占癌症死亡病例的18.0%。大多数患者在发现肺癌时已属中晚期，无法接受手术治疗，诊断后的5年生存率仅为10%-20%^[1]^。低剂量螺旋计算机断层扫描（computed tomography, CT）可在早期诊断癌症，降低死亡风险。意大利多中心肺部检测结果^[2]^显示，与未进行低剂量CT筛查相比，低剂量CT筛查组在第10年总死亡率降低20%。不同病理类型肺癌治疗方式不同，非小细胞肺癌（non-small cell lung cancer, NSCLC）患者，根据其基础身体状况、年龄及临床病理分期等因素选择治疗方式，通常包括手术、放化疗、分子靶向治疗、血管靶向治疗等；小细胞肺癌（small cell lung cancer, SCLC）好发于吸烟且有家族史的成年男性，早期及局部晚期患者一线治疗推荐依托泊苷+铂类化疗联合放疗^[3]^。虽然治疗方式众多，但仍有部分患者出现治疗后进展或复发，预后较差。近年来，随着免疫治疗的加入，肺癌患者的总生存状况得到明显改善。免疫联合或单药应用，无论在一线还是后线治疗都取得了突破性进展。其中阻断程序性死亡受体1（programmed cell death 1, PD-1）/程序性死亡配体1（programmed cell death ligand 1, PD-L1）通路等的多种抗体药物已应用于肺癌的临床治疗^[4]^。然而由于免疫耐药的发生^[5, 6]^，只有45.2%的患者符合免疫治疗的用药标准且从中获益^[7, 8]^。因此识别作用于不同通路或相同通路不同位点的其他免疫靶点分子极具研究前景。T细胞免疫球蛋白和免疫受体酪氨酸抑制基序结构域（T cell immunoreceptor with immunoglobulin and immunoreceptor tyrosine-based inhibition motif domain, TIGIT）作为新兴免疫检查点分子在2009年被三个不同的实验室通过基因组分析独立发现，而后受到广泛关注^[9]^。本文就TIGIT的结构、TIGIT对免疫细胞的作用及在肺癌治疗中的研究进展等作一综述。

## 1 TIGIT及其配体结构

### 1.1 TIGIT结构

TIGIT全称为T细胞免疫球蛋白和免疫受体酪氨酸抑制基序结构域，又名华盛顿大学细胞黏附分子（Washington University cell adhesion molecule, WUCAM）、V-集和跨膜结构域含蛋白3（V-set and transmembrane do-main-containing protein 3, Vstm3）或V-集和含免疫球蛋白结构域蛋白9（V-set and immunoglobulin domain-containing protein 9, VSIG9），从属于脊髓灰质炎病毒受体结合素家族，该家族为免疫球蛋白超家族的一员^[9, 10]^。其分子结构包括1个细胞外免疫球蛋白可变集结构域（IgV）、1个I型跨膜结构域以及1个具有经典免疫受体酪氨酸基抑制基序（immunoreceptor tyrosine-based inhibitory motif, ITIM）和免疫球蛋白酪氨酸尾（immunoglobulin tyrosine tail, ITT）基序的细胞内结构域，其中ITT基序在肿瘤免疫抑制中起主要作用。TIGIT高表达于活化的CD4^+^ T和CD8^+^ T细胞、自然杀伤（natural killer, NK）细胞、调节性T细胞（regulatory T cells, Tregs）和滤泡辅助性T细胞（follicular helper T cells, Tfh）表面^[11-13]^，与共刺激分子DNAX辅助分子1（DNAX accessory molecule 1, DNAM-1 CD226）竞争性与脊髓灰质炎病毒受体（poliovirus receptor, PVR）等配体结合传递免疫抑制的信号，通过诱导调节免疫细胞功能，帮助肿瘤实现免疫逃逸。

### 1.2 TIGIT相关配体结构（图 1）

**图 1:**
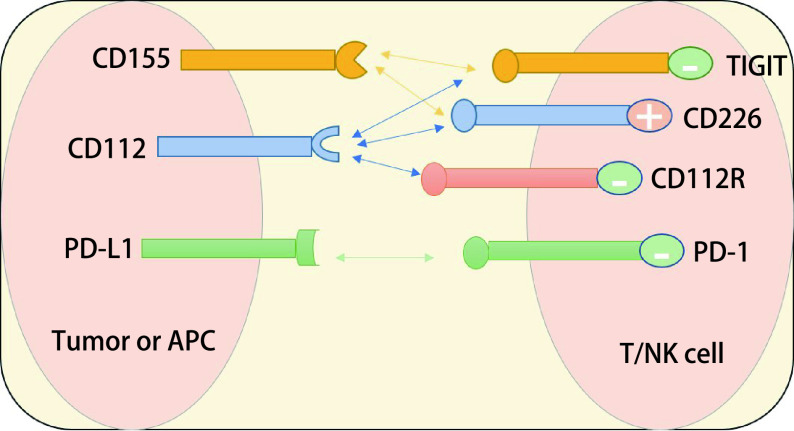
TIGIT及其配体示意图。PD-1/PD-L1结合发挥免疫抑制作用；TIGIT可与CD155及CD112等配体结合；TIGIT为共抑制受体，发挥免疫抑制作用；CD226为共刺激受体。 Diagram of TIGIT and its ligand. PD-1/PD-L1 binding plays an immunosuppressive role; TIGIT can bind to the ligands of CD155 and CD112. TIGIT is a co-inhibitory receptor, which has immunosuppressive effect. CD226 is a costimulatory receptor. TIGIT: T cell immunoreceptor with immunoglobulin and immunoreceptor tyrosine-based inhibition motif domain; PD-1: programmed cell death 1; PD-L1: programmed cell death ligand 1; APC: antigen presenting cell; NK: natural killer; CD: cluster of differentiation.

TIGIT的主要配体包括三种，白细胞分化抗原155（cluster of differentiation 155, CD155）、白细胞分化抗原112（cluster of differentiation 112, CD112）及白细胞分化抗原113（cluster of differentiation 113, CD113）^[14]^。其中CD155是在人和小鼠中与TIGIT亲和力最高的同源配体，又被称为PVR、柄蛋白样蛋白5（stalk protein-like protein 5, Necl5）和肿瘤相关糖蛋白E4（tumor-associated glycoprotein E4, Tage4）。它是一种跨膜黏附蛋白，属免疫球蛋白超家族，由跨膜区、具有三个Ig样环的细胞外区域和胞质区域组成。CD155表达于肿瘤细胞或抗原提呈细胞表面，通过促进细胞的增殖和迁移对肿瘤的发生发展产生重要作用，在非免疫细胞如肾脏、肺、胰腺等细胞表面也有少量表达。Oyama等^[15]^的研究显示CD155高表达的患者更易出现肺癌的侵袭转移，CD155阳性的患者PD-L1抑制剂治疗效果较PD-L1阴性患者差，且CD155高表达预示了患者预后不良。CD112，又被称为PVRL2、nectin-2，表达于抗原提呈细胞或肿瘤细胞的表面，在造血系统细胞和非造血系统系统细胞表面均广泛表达，是nectin家族成员之一。CD112与白细胞分化抗原112受体（cluster of differentiation 112 receptor, CD112R）或TIGIT结合抑制T细胞增殖。CD113仅在非造血系统细胞表面表达。TIGIT与CD112及CD113亲和力均较弱^[16, 17]^。

## 2 TIGIT对免疫细胞的作用机制

在肿瘤理想的发生发展过程中，肿瘤细胞坏死释放肿瘤抗原，肿瘤抗原被树突状细胞摄取并迁移至淋巴结传递给T细胞引起初始T细胞活化，活化的T细胞进入循环系统发挥免疫效应。但在实际状态下，肿瘤细胞会产生及诱导一系列的变化来实现肿瘤免疫逃逸。

### 2.1 TIGIT在T细胞中的抑制机制

白细胞介素-10（interleukin-10, IL-10）是一种抗炎细胞因子，促进T细胞衰竭及免疫抑制的发生^[9]^。TIGIT可通过与CD155结合，促进*Src*基因（sarcoma gene, *Src*）家族激酶Fyn和Lck的磷酸化，募集含Src同源性2结构域的蛋白酪氨酸磷酸酶1（Src homology 2 domain-containing protein tyrosine phosphatase 1, SHP-1）磷酸酶来下调与免疫细胞功能相关的PI3K、MAPK、核因子κB（nuclear factor kappa B, NF-κB）通路，抑制细胞因子的产生，从而发挥CD4^+^ T细胞的内在抑制作用^[11, 18]^。以往的研究^[9, 19]^认为TIGIT通过与抗原提呈细胞（antigen presenting cell, APC）（如树突状细胞）上的CD155相结合，抑制了APC的共刺激能力，导致肿瘤抗原提呈减少，从而增加了IL-10的分泌，减少了IL-12的分泌，间接对T细胞的功能产生了抑制作用。Solomon等^[20]^发现，TIGIT可与肿瘤细胞上的CD155直接结合，触发T细胞的直接抑制信号，实现免疫逃逸。TIGIT还可损害CD226同源二聚化的能力并以更高的亲和力阻止CD226与CD115结合^[21, 22]^，从而阻碍CD226介导的T细胞活化。TIGIT在效应T细胞中表达上调抑制了免疫反应，而在Tregs细胞的表达上调则促进了免疫抑制作用^[23]^。有研究^[24]^认为，TIGIT主要通过Tregs调节免疫功能。TIGIT+Tregs细胞可上调共抑制受体TIM-3的表达，两者协同抑制肿瘤的免疫反应。同时，TIGIT位点去甲基化水平增强及其与配体结合会引起纤维原样蛋白2（fibrinogen-like protein 2, Fgl2）表达，从而作用于T细胞产生抑制作用^[25]^。研究^[21, 24, 26-28]^表明，TIGIT在CD8^+^ T细胞及Treg细胞上表达升高预示着患者预后更差。

### 2.2 TIGIT在NK细胞中的抑制机制

TIGIT通过抑制肿瘤细胞初始死亡及释放抗原来抑制NK细胞效应^[10, 29, 30]^。活化的NK细胞可合成并分泌多种细胞因子，发挥直接杀伤靶细胞的作用，其杀伤活性与细胞内嗜天青颗粒的含量呈正相关。Stanietsky等^[10]^最早在2009年发现了TIGIT通过其ITIM基序介导NK细胞上的抑制信号，抑制NK细胞脱颗粒、细胞因子分泌^[31]^及直接抑制NK细胞介导的细胞毒作用。此外，来自肠道具核梭杆菌的梭杆菌自动转动蛋白2（fusobacterium autotransporter protein 2, Fap2）与TIGIT直接结合，可触发抑制NK细胞的信号。在血液系统肿瘤和实体肿瘤相关实验^[30, 32]^中均发现，TIGIT高表达与NK细胞耗竭相关，削弱了NK细胞介导的肿瘤免疫。在荷瘤小鼠模型中^[30]^，阻断TIGIT可以抑制NK细胞的耗竭，增强NK细胞的抗肿瘤活性，促进NK细胞的免疫功能。

## 3 TIGIT在肺癌的研究进展

Yang等^[33]^的研究发现，在肺鳞癌的免疫组化结果中，CD155阳性率远高于PD-L1，这提示TIGIT/CD155轴在肺鳞癌的发生发展过程中起到了重要的作用。TIGIT的高表达与肺腺癌的高恶性程度相关^[34]^。过表达CD155的肺腺癌及SCLC患者的无进展生存期（progression-free survival, PFS）及总生存期（overall survival, OS）更短^[34-36]^，尤其是在PD-L1阳性患者中这一趋势更为明显^[15]^。表明CD155及TIGIT可用来预测临床结局，实现肺癌个性化治疗。目前，TIGIT抑制剂单药或联合PD-1/PD-L1抑制剂治疗肺癌的临床试验层出不穷（表 1）。针对TIGIT抑制剂替瑞利尤单抗（Tiragolumab）联合PD-L1抑制剂阿替利珠单抗（Atezolizumab）治疗肺癌的II期临床研究-CITYSCAPE研究最为经典^[37]^。该研究纳入PD-L1≥1%的晚期NSCLC患者，根据PD-L1是否≥50%进行分层，分别以双免疫、安慰剂+Atezolizumab进行患者随机分组。主要终点为客观缓解率和PFS；主要的次要终点是反应持续时间、OS和安全性。结果显示与安慰剂+Atezolizumab组相比，双免疫组客观缓解率及PFS的改善情况均具有临床意义，且PD-L1高表达组获益更明显。然而遗憾的是，后续针对广泛期SCLC的III期临床研究SKYSCRAPER-02已宣告失败，针对PD-L1高表达的NSCLC患者的III期临床研究SKYSCRAPER-01的PFS未达到阳性结果，OS暂未出。至此，联合TIGIT及PD-1/PD-L1抑制剂治疗肺癌的临床价值受到严峻挑战。然而，多项研究发现TIGIT/CD155与PD-1/PD-L1共表达，且两者表达呈现正相关^[34, 38]^。联合抑制TIGIT与PD-1/PD-L1较单药肿瘤抑制效果更佳^[22, 39, 40]^。这表明在理论上双免疫治疗肺癌是切实可行的。针对临床试验失败的原因，首先从目标患者来说，选择的人群为PD-L1高表达患者，在一定程度上忽略了TIGIT的表达情况，可能最终对药物疗效造成影响；从机制研究来说，阻断TIGIT及PD-1/PD-L1可减少共抑制受体的肿瘤免疫逃逸，但是T细胞及NK细胞的激活通路是否恢复如初，需要进一步研究进行验证。维博利单抗（Vibostolimab）单药或与派姆单抗（Pembrolizumab）联合治疗晚期NSCLC在I期临床试验得出两者联合可表现出很好的抗肿瘤活性的结果^[41]^。从药物安全性方面来说，TIGIT相关的临床试验均表现出较好的药物安全性，例如在CITYSCAPE研究中，3级-4级治疗相关不良事件与3级-4级免疫相关不良事件发生频率在免疫单药及免疫联合组均相似，这提示TIGIT可能相较于PD-1/PD-L1通路是更下游的抑制位点，因此TIGIT抑制剂的应用在免疫不良反应方面可能弱于PD-1/PD-L1抑制剂。

**表 1:** 肺癌相关的TIGIT临床试验 Clinical trials of TIGIT associated with lung cancer

Research	Identifier	Medicine	Clinical phase	Target population	Intervening measure	Primary endpoint	Sponsor
KeyMaker-U01A	NCT04165070	Vibostolimab	II	Advanced NSCLC (treatment-naive)	Pembrolizumab+Chemotherapy+ Vibostolimab or MK4830 or MK-5890±Pemetrexed (NSQ)	ORR	Merck Sharp & Dohme
KEYVIBE-002	NCT04725188	MK-7684A (Vibostolimab+Pembrolizumab)	II	Stage IV metastatic NSCLC (after treatment)	MK-7684A±Docetaxel *vs* Docetaxel	PFS	Merck Sharp & Dohme
KEYVIBE -003	NCT04738487	MK-7684A (Vibostolimab+Pembrolizumab)	III	PD-L1 (+) metastatic NSCLC	MK-7684A *vs* Pembrolizumab	PFS; OS	Merck Sharp & Dohme
KEYVIBE -006	NCT05298423	MK-7684A (Vibostolimab+Pembrolizumab)	III	Stage III NSCLC	MK-7684A+CCRT followed by MK-7684A *vs* CCRT followed by Durvalumab	PFS; OS	Merck Sharp & Dohme
KEYVIBE -007	NCT05226598	MK-7684A (Vibostolimab+Pembrolizumab)	III	Metastatic NSCLC	MK-7684A+Chemotherapy *vs* Pembrolizumab+Chemotherapy	PFS; OS	Merck Sharp & Dohme
KEYVIBE -008	NCT05224141	MK-7684A (Vibostolimab+Pembrolizumab)	III	ES-SCLC (treatment-naive)	MK-7684A+Chemotherapy *vs* Atezolizumab+Chemotherapy	OS	Merck Sharp & Dohme
SKYROCKET	NCT05034055	Tiragolumab	II	Metastatic NSCLC (treatment-naive)	SBRT followed by Atezolizumab/Tiragolumab	PFS	Yonsei University
SKYSCRAPER-01	NCT04294810	Tiragolumab	III	Previously untreated locally advanced unresectable or metastatic PD-L1-selected NSCLC	Atezolizumab+Tiragolumab *vs* Atezolizumab+Placebo	PFS; OS	Hoffmann-La Roche
SKYSCRAPER-02	NCT04256421	Tiragolumab	III	Untreated extensive-stage SCLC	Tiragolumab+Atezolizumab +Chemotherapy *vs* Placebo+ Atezolizumab+Chemotherapy	PFS; OS	Hoffmann-La Roche
SKYSCRAPER-02C	NCT04665856	Tiragolumab	III	Untreated extensive-stage SCLC	Tiragolumab+Atezolizumab+ Chemotherapy *vs* Placebo+Atezolizumab+Chemotherapy	PFS/OS in the Primary Population	Hoffmann-La Roche
SKYSCRAPER-03	NCT04513925	Tiragolumab	III	Locally advanced, unresectable stage III NSCLC	Atezolizumab+Tiragolumab *vs* Durvalumab	PFS	Hoffmann-La Roche
SKYSCRAPER-06	NCT04619797	Tiragolumab	II/III	Previously untreated advanced NSQ	Tiragolumab+Atezolizumab+Chemotherapy *vs* Placebo+Pembrolizumab+ Chemotherapy	ORR; PFS; OS	Hoffmann-La Roche
GO40290	NCT03563716	Tiragolumab	II	Chemotherapy-naive patients with locally advanced or metastatic NSCLCL	Tiragolumab+Atezolizumab	ORR; PFS	Genentech
GO42501	NCT04832854	Tiragolumab	II	Previously untreated locally advanced resectable stage II, IIIA, or select IIIB NSCLC	Neoadjuvant and adjuvant Tiragolumab+Atezolizumab	Number of participants with surgical delays, operative and post-operative complications, surgical cancellations related to study treatment; Percentage of participants with adverse events; MPR	Hoffmann-La Roche
ML41257	NCT04308785	Tiragolumab	II	LS-SCLC	CRT followed by Atezolizumab+Tiragolumab *vs* CRT followed by Atezolizumab+Placebo	PFS of ITT population	Hoffmann-La Roche
AdvanTIG-204	NCT04952597	Ociperlimab	II	Untreated limited-stage SCLC	Ociperlimab+Tislelizumab+ Chemoradiotherapy	PFS	BeiGene
AdvanTIG-205	NCT05014815	Ociperlimab	II	Untreated metastatic NSCLC	Ociperlimab+Tislelizumab+Chemotherapy	PFS	BeiGene
AdvanTIG-301	NCT04866017	Ociperlimab	III	Previously untreated, locally advanced, unresectable NSCLC	Ociperlimab+Tislelizumab+CCRT followed by Ociperlimab+Tislelizumab *vs* Tislelizumab+CCRT followed by Tislelizumab *vs* CCRT followed by Durvalumab	PFS/CRR of ITT	BeiGene
AdvanTIG-302	NCT04746924	Ociperlimab	III	Untreated lung cancer	Tislelizumab+Ociperlimab (Arm A) *vs* Pembrolizumab+Placebo (Arm B) *vs* Tislelizumab+Placebo (Arm C)	PFS; OS	BeiGene
ARC-7	NCT04262856	Domvanalimab (AB-154)	II	PD-L1 positive NSCLC	Zimberelimab *vs* Zimberelimab+Domvanalimab *vs* Zimberelimab+Domvanalimab+ Etrumadenant	ORR; PFS	Arcus Biosciences
STAR-121	NCT05502237	Domvanalimab (AB-154)	III	Untreated metastatic NSCLC	Zimberelimab+Domvanalimab+Chemotherapy *vs* Pembrolizumab+Chemotherapy	PFS; OS	Gilead Sciences
ARC-10	NCT04736173	Domvanalimab (AB-154)	III	PD-L1 positive NSCLC	Chemotherapy (Arm A) *vs* Zimberelimab (Arm B) *vs* Zimberelimab+Domvanalimab (Arm C)	OS (Arm A *vs* Arm B, Arm B *vs* Arm C); PFS (Arm B *vs* Arm C)	Arcus Biosciences
PACIFIC-8	NCT05211895	Domvanalimab (AB-154)	III	Stage III unresectable NSCLC	Durvalumab+Domvanalimab *vs* Durvalumab+Placebo	PFS	Arcus Biosciences
CA020-016	NCT05005273	BMS-986207	II	Stage IV NSCLC	Nivolumab+Ipilimumab+BMS-986207 *vs* Nivolumab+Ipilimumab+Placebo	PFS	Bristol-Myers Squibb
Landscape 1011 study	Landscape 1011 study	SEA-TGT	I/II	Advanced NSCLC	Immunotherapy (Sasanlimab) in combination with targeted therapies	DLT; DORR; ORR	Pfizer
213824	213824	EOS-448	II	PD-L1 high (Tumor cells/tumor proportion score≥50%), previously untreated, unresectable, locally advanced or metastatic NSCLC	GSK4428859A (Pembrolizumab+Dostarlimab)	ORR	Dostarlimab
CIBI939A102 (Ia)	NCT04672356	IBI-939	I	Advanced lung cancer	IBI939+Sintilimab	Adverse events; RP2D	Innovent Biologics (Suzhou) Co.
CIBI939A102 (Ib)	CIBI939A102 (Ib)	IBI-939	I	Advanced lung cancer	IBI939+Sintilimab	ORR	Innovent Biologics (Suzhou) Co.
NSCLC: non-small cell lung cancer; NSQ: advanced non-squamous non-small cell lung cancer; ORR: objective response rate; PFS: progression-free survival; OS: overall survival; CCRT: concurrent radiochemotherapy; ES-SCLC: extensive small cell lung cancer; SBRT: stereotactic ablative radiotherapy; MPR: major pathological response; ITT: intent-to-treat; CRR: complete response rate; CRT: radiochemotherapy; DLT: dose-limiting toxicities; DORR: durable objective response rate; RP2D: recommended phase II dose.							

## 4 TIGIT表达与其他免疫靶点的关系

TIGIT与其他免疫检查点表达，尤其PD-1/PD-L1表达之间存在联系。PD-1为I型膜蛋白，结构包括胞外IgV结构域，跨膜区和包含ITIM的胞内结构，其表达于活化的T细胞、B细胞及巨噬细胞表面，可广泛地对免疫反应进行负性调节。Banta等^[22]^研究发现，TIGIT及PD-1抑制剂的疗效与CD226的表达有关，TIGIT及PD-1各自独立调节CD226的表达情况。PD-1通过其胞内的ITIM结构域抑制CD226磷酸化，TIGIT通过胞外结构与CD155结合直接抑制CD226传递共刺激信号^[14]^。因此，同时阻断TIGIT及PD-1可使CD226恢复共刺激作用，增强CD8^+^ T细胞的抗肿瘤作用。这一点在黑色素瘤患者及结肠癌小鼠模型中均已得到证实^[21, 26]^。此外，Kurtulus等^[24]^研究发现，TIGIT表达于Tregs细胞可上调共抑制受体T细胞免疫球蛋白黏蛋白分子3（T cell immunoglobulin mucin molecule 3, TIM-3）的表达，两者表现出协同作用，共同实现肿瘤免疫逃逸。

## 5 TIGIT在其他类型肿瘤中的研究

Guillerey等^[42]^研究表明，在骨髓瘤患者中CD8^+^ T细胞比CD4^+^ T细胞和NK细胞表达更高水平的TIGIT。黑色素瘤、乳腺癌、胃癌、结直肠癌、肾透明细胞癌等实体肿瘤中均发现肿瘤组织的TIGIT表达高于癌旁正常组织^[43]^，且TIGIT、PD-1的高表达与长生存呈现出负相关^[44, 45]^。在结肠癌小鼠模型中^[21]^同时阻断TIGIT/PD-L1与单独阻断一种免疫检查点相比，肿瘤体积减少更明显，这说明联合阻断TIGIT、PD-1，可逆转细胞毒性T淋巴细胞（cytotoxic T lymphocyte, CTL）的效应，进一步抑制肿瘤的发生发展，阻止免疫逃逸的发生，未来免疫抑制剂的联合应用是肿瘤免疫治疗的趋势。

## 6 总结与展望

综上所述，TIGIT是一种抑制性免疫检查点，通过与CD155等抗体结合，促进NK细胞耗竭、减少细胞因子的分泌，还可通过直接或间接作用抑制效应T细胞、上调Tregs细胞的作用，发挥免疫抑制功能。TIGIT与PD-1/PD-L1、TIM-3等共抑制受体发挥协同抑制作用。在多种实体瘤及血液系统肿瘤中发现，同时抑制PD-1/PD-L1及TIGIT较单独抑制获得更佳的结果，但抑制的顺序是否与肿瘤抑制效果有关，亟待进一步实验进行验证。此外，如何在抑制TGIT信号通路的基础上激活并上调CD226等刺激性信号通路的表达，也期待后续更大规模实验的验证。
